# Chemical Constituents from Ethanoic Extracts of the Aerial Parts of *Leea aequata* L., a Traditional Folk Medicine of Myanmar

**DOI:** 10.1007/s13659-019-0209-y

**Published:** 2019-05-07

**Authors:** Nay Lin Tun, Dong-Bao Hu, Meng-Yuan Xia, Dong-Dong Zhang, Jun Yang, Thaung Naing Oo, Yue-Hu Wang, Xue-Fei Yang

**Affiliations:** 1 Southeast Asia Biodiversity Research Institute, Chinese Academy of Sciences, Yezin, Nay Pyi Taw, 05282 Myanmar; 2 Key Laboratory of Economic Plants and Biotechnology and the Yunnan Key Laboratory for Wild Plant Resources, Kunming Institute of Botany, Chinese Academy of Sciences, Kunming, 650201 People’s Republic of China; 3 University of Chinese Academy of Sciences, Beijing, 100049 People’s Republic of China; 4 College of Resources and Environment, Yuxi Normal University, Yuxi, 653100 People’s Republic of China; 5 Forest Research Institute, Yezin, Nay Pyi Taw, 05282 Myanmar

**Keywords:** *Leea aequata*, Vitaceae, Traditional medicines, Lignans, Flavonoids

## Abstract

**Abstract:**

We aimed at reporting the chemical constituents and antimicrobial activities of *Leea aequata* L., a traditional folk medicine used in Myanmar for the treatment of wounds and skin diseases. A new neolignan, (7*S*,8*R*)-9′-*O*-acetylcedrusin (**1**), a new lactam, (3*S*,4*S*)-4-chloro-3-hydroxypiperidin-2-one (**2**), along with 21 known compounds, including five lignans (**3**–**7**), four flavonoid glycosides (**8**–**11**), and others (**12**–**23**), were isolated from the ethanoic extract of the aerial parts of *L. aequata*. The structures of the new compounds were determined by NMR, MS, and ECD spectra. For all the antimicrobial tests of the 23 compounds, only 3,4,5-trihydroxybenzoic acid ethyl ester (**17**) showed weak inhibitory activities against *Escherichia coli* and *Salmonella enterica* subsp. *enterica*.

**Graphical Abstract:**

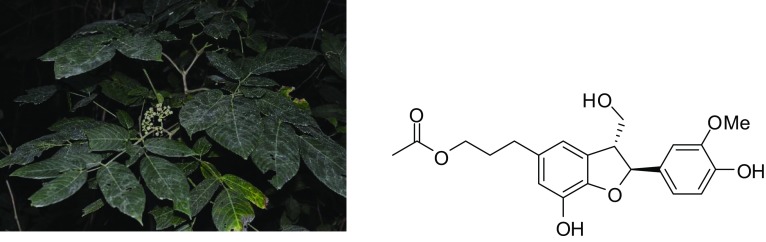

**Electronic supplementary material:**

The online version of this article (10.1007/s13659-019-0209-y) contains supplementary material, which is available to authorized users.

## Introduction

Medicinal plants and their traditional knowledge are important source for modern drug development. In Myanmar, majority of the populations had been relying on traditional herbal remedy for treatment of various diseases for generations. However, in the past several decades, Myanmar is behind the development of science and technology due to political unease and backward of social economic development, which kept the valuable knowledge and resources on medicinal plants un-known to the world; and thus rarely applied at international scale. Recently, Defilipps and Krupnick summarized the medicinal plants used in Myanmar, showing a total number of 472 plant species from 123 families used as herbal medicine [1]. *Medicinal Plant List of Myanmar*, a book published by FAME Company in Myanmar, recorded more than 1500 medicinal plant species [2]. Few ethnobotanical studies documented various list of medicinal plants used locally such as in southern Shan State [3], at Natma Taung National Park [4], and in southern Chin State [5]. These publications provide pilot investigations and fundamental information to understand the value of these wealthy biodiversity and culture for medicinal plants in Myanmar. Apart from these, applying modern technology, such as phytochemistry and pharmacology for Myanmar medicinal plant research were also surging. For example, Nwet Nwet Win published a series of papers on natural compounds isolated from *Kaempferia pulchra* [6], *Premna integrifolia* [7], and *Kayea assamica* [8, 9]. Other examples include extracts with anti-influenza virus property from *Jatropha multifida* [10], along with evaluation of antioxidant and antimicrobial activities of indigenous medicinal plants [11, 12]. However, the discovery of the traditional knowledge, biological constituents, and pharmacological properties of the vast pool of Myanmar medicinal plants just commences, and intensive field explorations and scientific validations are much needed.

The genus *Leea* belongs to the family of Vitaceae. Species of *Leea* are distributed from Africa to Asia, northeastern Australia, New Guinea and islands of the Pacific (Fiji, Solomon Islands, Caroline Islands) [13]. Some species are used as traditional folk medicines. For example, the roots of *L. asiatica* (L.) Ridsdale are used to treat icteric hepatitis in China [14], the roots of *L. macrophylla* Roxb. ex Hornem are used in medication for guineaworm in Myanmar [1], the leaves of *L. guineense* G. Don are used against cancers in Guinea [15], and the roots of *L. thorelii* Gagnep. are used as a tonic in Thailand [16]. Flavanoids and flavanoid glycosides are found to be the major constituents of the genus [16, 17].

*Leea aequata* is usually a shrub, less often a small tree, distributed in Bangladesh, Bhutan, Cambodia, China, India, Malaysia, Myanmar, Nepal, Philippines, Thailand, and Vietnam [18]. A previous research showed that the seeds, stems, and roots of *L. aequata* have antibacterial activity [19]. However, no knowledge is available on the chemical constituents of this species. In Mandalay, Myanmar, it is locally known as Kya-petthein (naga-mauk). The fresh leaves of the plant are crushed and externally used for treating wounds and skin diseases by Bamar people. During our field visit for inventory of medicinal plants in central Myanmar in Dec 2015, we collected the specimen of *L. aequata* and documented the traditional uses by local people around Myingyan, Kyaukpadaung, Po-pa Mountain in Mandalay. In Feb 2018, we continued a further investigation of ethnobotanical knowledge and collected the aerial parts of the species from the same site for phytochemical analysis. We aimed at isolating and understanding the chemical constituents and at testing the antimicrobial activities of this species.

## Results and Discussion

### Structure Elucidation

**Fig. 1:**
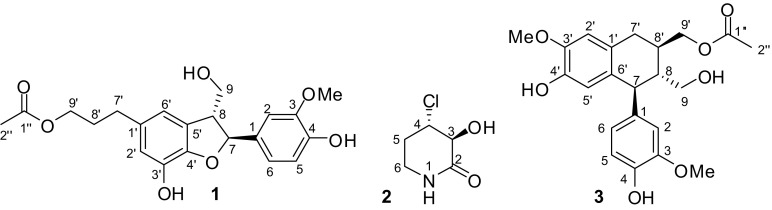
Chemical structures of compounds **1**–**3** from *Leea aequata*

Two undescribed compounds (**1** and **2**, Fig. 1) and 21 known compounds (**3**–**23**) were isolated from the ethanoic extracts of *L. aequata* by silica gel and Sephadex LH-20 column chromatography and semipreparative HPLC.

**Fig. 2:**
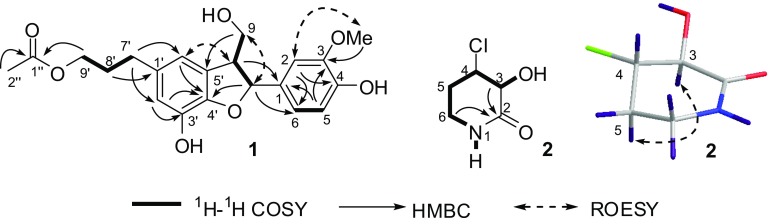
Key 2D NMR correlations of compounds **1** and **2**

Compound **1** was found to possess the molecular formula of C_21_H_24_O_7_ by ^13^C NMR data (Table 1) and HRESIMS at *m/z* 411.1415 [M + Na]^+^ (calcd for C_21_H_24_NaO_7_, 411.1420). Its NMR data (Table 1) indicated the presence of one 1,2,4-trisubstituted phenyl ring [*δ*_H_ 7.00 (d, *J* = 1.8 Hz), 6.86 (dd, *J* = 8.1, 1.8 Hz), and 6.78 (d, *J* = 8.1 Hz)], one 1,2,3,5-tetrasubstituted phenyl ring [*δ*_H_ 6.62 (br s) and 6.58 (d, *J* = 1.2 Hz)], an acetyl group [*δ*_C_ 173.1 and 20.8; *δ*_H_ 2.04 (3H, s)], one methoxy group [*δ*_C_ 56.4; *δ*_H_ 3.84 (3H, s)], four *sp*^3^ methylenes, and two *sp*^3^ methines, which implied that compound **1** might be an acetylated neolignan. By comparing its NMR data with those of cedrusin [20], **1** was deduced to be an acetylated derivative of cedrusin, which was confirmed by COSY and HMBC correlations (Fig. 2). The acetoxyl group was located at C-9′ based on the HMBC correlations from H_2_-9′ to C-1″. The *trans* relationship between H-7 and H-8 was elucidated by the ROESY correlations of H-7/H_2_-9 (Fig. 2), as well as the chemical shift of C-8 (*δ*_C_ 55.7) [21]. In this type of neolignans, the chemical shift of C-8 is approximately 54 ppm in the *trans* isomers and is approximately 49 ppm in the *cis* analogues [21]. The absolute configurations of dihydrobenzofuran neolignans are usually determined by the signs of the band ^1^L_b_ (270–300 nm) or ^1^L_a_ (220–240 nm) in their ECD spectra. The positive signs of the ^1^L_b_ band in the ECD spectra predict the absolute configuration of 7,8-*trans*-3-methoxydihydrobenzofuran neolignans to be 7*S*,8*R* [21–23]. The ECD spectrum of **1** showed a positive Cotton effect at 294 nm (Δ*ε* + 0.52). Therefore, the structure of **1** was determined to be (7*S*,8*R*)-9′-*O*-acetylcedrusin.

**Table 1:** ^1^H (800 MHz) and ^13^C NMR (200 MHz) data of **1** and **3** in CD_3_OD

No.	**1**		**3**	
	*δ*_H_ (*J*, Hz)	*δ*_C_	*δ*_H_ (*J*, Hz)	*δ*_C_
1		135.1		138.4
2	7.00 (d, 1.8)	110.5	6.68 (d, 2.0)	113.9
3		149.1		149.0
4		147.4		146.0
5	6.78 (d, 8.1)	116.1	6.73 (d, 8.0)	116.0
6	6.86 (dd, 8.1, 1.8)	119.7	6.60 (dd, 8.0, 2.0)	123.2
7	5.51 (d, 6.1)	88.7	3.86 (d, 10.1)	47.6
8	3.47 (m)	55.7	1.79 (m)	47.5
9	3.84 (m)	65.1	3.60 (dd, 11.6, 3.3)	61.6
	3.77 (dd, 11.1, 7.3)		3.39 (dd, 11.6, 3.6)	
1′		135.9		128.4
2′	6.58 (d, 1.2)	117.0	6.65 (s)	112.3
3′		142.0		147.3
4′		146.7		145.5
5′		129.9	6.19 (s)	117.4
6′	6.62 (br s)	116.7		133.9
7′	2.60 (2H, dd, 7.9, 7.1)	32.8	2.82 (dd, 15.8, 4.7)	33.5
			2.75 (dd, 15.8, 10.5)	
8′	1.92 (2H, m)	31.7	2.22 (m)	36.7
9′	4.07 (2H, td, 6.6, 1.0)	65.1	4.26 (dd, 11.0, 4.2)	68.2
			4.11 (dd, 11.0, 7.2)	
1″		173.1		173.2
2″	2.04 (3H, s)	20.8	2.04 (3H, s)	20.8
3-OMe	3.84 (3H, s)	56.4	3.78 (3H, s)	56.4
3′-OMe			3.80 (3H, s)	56.4

**Fig. 3:**
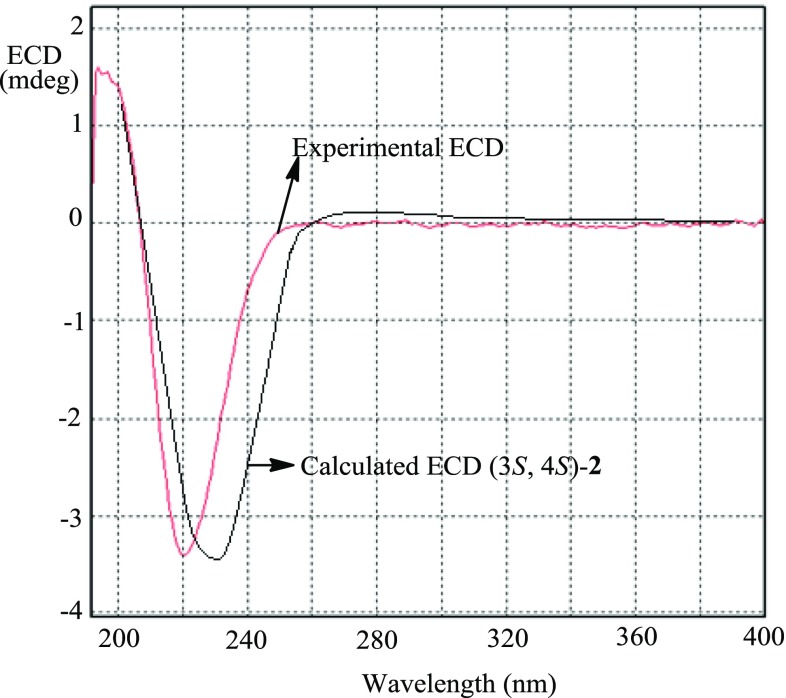
Experimental and calculated ECD for compound **2**

Based on ^13^C NMR data (Table 2) and HRESIMS with a positive ion at *m/z* 172.0135 [M + Na]^+^ (calcd for C_5_H_8_ClNNaO_2_, 172.0141), the molecular formula of **2** was deduced to be C_5_H_8_ClNO_2_. The ^13^C NMR spectrum of **2** indicated five signals for a carbonyl group, two *sp*^3^ methylenes, and two *sp*^3^ methines. According to COSY correlations (Fig. 2), a carbon connection from C-3 to C-6 was confirmed. Based on the HMBC correlations (Fig. 2) from H-6 to C-2, compound **2** was deduced to be a lactam. 3-Hydroxy substitution was elucidated by the COSY correlation of H-3/3-OH. The remaining chlorine atom must attach to the last methine group (C-4). In order to elucidate the relative configuration of **2**, H-3 was assumed to be α-oriented. The *trans* configuration of H-3 and H-4 was elucidated by the *J*_3,4_ value (7.7 Hz) and the ROESY correlation of H-3/H-5α, which indicated that both H-3 and H-5α (*δ*_H_ 2.02) were axial and α-oriented, while H-4 was axial and β-oriented. The absolute configuration of **2** was established as 3*S*,4*S* by comparison of the experimental and calculated ECD (Fig. 3). Thus, the structure of **2** was determined to be (3*S*,4*S*)-4-chloro-3-hydroxypiperidin-2-one.

**Table 2:** ^1^H (500 MHz) and ^13^C NMR (126 MHz) data of **2**

No.	**2** in DMSO-*d*_6_		**2** in CD_3_OD)	
	*δ*_H_ (*J*, Hz)	*δ*_C_	*δ*_H_ (*J*, Hz)	*δ*_C_
2		169.5		172.5
3	3.78 (br d, 7.7)	72.8	3.99 (d, 7.5)	74.5
4	4.20 (ddd, 9.8, 7.7, 3.5)	60.1	4.17 (ddd, 9.6, 7.5, 3.5)	56.0
5β	2.23 (m)	29.2	2.38 (m)	30.3
5α	2.02 (m)		2.12 (m)	
6	3.15 (2H, m)	37.9	3.34 (2H, m)	39.6
3-OH	5.78 (br s)			
NH	7.68 (br s)

9′-*O*-Acetylisolariciresinol (**3**) was previously reported without NMR data, which are presented in the paper (Table 1). Other known compounds, 9-*O*-acetylisolariciresinol (**4**) [24], (+)-lariciresinol (**5**) [25], (+)-syringaresinol (**6**) [26], urolignoside (**7**) [27], astragalin (**8**) [28], isorhamnetin 3-*O*-β-d-glucopyranoside (**9**) [29], isoquercitrin (**10**) [30], mauritianin (**11**) [31], *trans*-*N*-*p*-coumaroyltyramine (**12**) [32], *N*-*trans*-feruloyltyramine (**13**) [33], vanillic acid (**14**) [34], syringic acid (**15**) [35], α-hydroxyacetovanillone (**16**) [36], 3,4,5-trihydroxybenzoic acid ethyl ester (**17**) [37], dihydro-*p*-methoxy cinnamic acid (**18**) [38], isotachioside (**19**) [39], (6*S*,9*S*)-roseoside C (**20**) [40], (6*S*,9*R*)-roseoside (**21**) [40], scopoletin (**22**) [41], and 5-hydroxymethylfurfural (**23**) [42], were determined by comparing their spectroscopic data with those reported in the literature.

### Antimicrobial Assay

All compounds (**1**–**23**) from the plants were measured for antimicrobial activities against four bacteria, *Escherichia coli*, *Staphylococcus aureus* subsp*. aureus*, *Salmonella enterica* subsp. *enterica*, and *Pseudomonas aeruginosa*, along with one fungus *Candida albicans*. 3,4,5-Trihydroxybenzoic acid ethyl ester (**17**) showed weak inhibitory activities against *E. coli* (43.8% inhibition) and *S. enterica* subsp*. enterica* (46.8% inhibition) at a concentration of 128 µg/mL. Inhibitions of other compounds were < 30%.

## Experimental Section

### General Experimental Procedures

Optical rotations were recorded using a JASCO P-1020 Polarimeter (Jasco Corp., Tokyo, Japan). Ultraviolet (UV) spectra were taken on a Shimadzu UV-2401 PC spectrophotometer (Shimadzu, Kyoto, Japan). Electronic circular dichroism (ECD) spectra were recorded on a Chirascan CD spectrometer (Applied Photophysics Ltd., Leatherhead, UK). ^1^H and ^13^C Nuclear magnetic resonance (NMR) spectra were collected on a Bruker AM-400, a Bruker DRX-500, a Bruker Avance III-600, and a Bruker Ascend™ 800 MHz spectrometers (Bruker Corp., Karlsruhe, Germany) with tetramethylsilane (TMS) as an internal standard. Electrospray ionization mass spectrometry (ESIMS) and high-resolution electrospray ionization mass spectrometry (HRESIMS) analyses were performed on an API QSTAR Pulsar 1 spectrometer (Applied Biosystems/MDS Sciex, Foster City, CA, USA). Silica gel G (80–100 and 300–400 mesh, Qingdao Meigao Chemical Co., Ltd., Qingdao, China), C_18_ silica gel (40–75 μm, Fuji Silysia Chemical Ltd., Aichi, Japan), and Sephadex LH-20 (GE Healthcare Bio-Sciences AB, Uppsala, Sweden) were used for column chromatography, and silica gel GF_254_ (Qingdao Meigao Chemical Co., Ltd.) was used for preparative thin layer chromatography (TLC) as precoated plates. TLC spots were visualized under UV light at 254 nm and by dipping into 5% H_2_SO_4_ in alcohol followed by heating. Semipreparative high-performance liquid chromatography (HPLC) was performed on an Agilent 1200 series pump (Agilent Technologies, Santa Clara, USA) equipped with a diode array detector, an Agilent Zorbax SB-C_18_ column (5.0 μm, *ϕ* 9.4 × 250 mm), and a Welch Ultimate AQ-C_18_ column (5.0 μm, *ϕ* 4.6 × 300 mm).

### Plant Material

The aerial parts of *L. aequata* were collected from Myingyan, Kyaukpadaung, Po-pa Mountain, Trekking trails near Po-pa mountain resort (20°55′05″N and 95°13′38″E), in Dec 2015, and identified by Dr. Jie Cai, at Kunming Institute of Botany, Chinese Academy of Sciences. A voucher specimen (No. 15CS10775) was deposited at the herbarium of the Forest Research Institute (FRI), Myanmar and the KUN, Kunming Institute of Botany, Chinese Academy of Sciences.

### Extraction and Isolation

The air-dried, powdered *L. aequata* plant (3.13 kg) was exhaustively extracted with EtOH (4 × 15 L) at room temperature for 3 days every time. The EtOH extracts (292 g) were suspended in H_2_O and further partitioned with petroleum ether, ethyl acetate, and *n*-butanol to yield petroleum ether-soluble (discarded), ethyl acetate-soluble (71.9 g, part A), and *n*-butanol-soluble parts (40.9 g, part B), respectively.

Part A was subjected to column chromatography (silica gel; petroleum ether-EtOAc, 20:1 → 0:1, v/v)) to yield two fractions (A1 and A2). Fraction A1 was separated on an RP-18 silica gel column eluted with MeOH–H_2_O (30% → 100%) to yield three main subfractions. The 30% MeOH-eluted portion was purified by Sephadex LH-20 column chromatography (MeOH) and semipreparative HPLC [Agilent Zorbax SB-C_18_ column, MeOH–H_2_O (containing 0.05% TFA), 30:70, 2 mL/min] to yield **14** (1.2 mg, *t*_R_ = 19.652 min) and **15** (2.5 mg, *t*_R_ = 22.485 min). The 50% MeOH-eluted portion was purified by Sephadex LH-20 column chromatography (MeOH) and semipreparative HPLC [Agilent Zorbax SB-C_18_ column, MeOH–H_2_O (containing 0.05% TFA), 25:75, 2 mL/min] to yield **17** (6.2 mg, *t*_R_ = 25.766 min) and **22** (3.1 mg, *t*_R_ = 43.296 min). The 60% MeOH-eluted portion was purified by Sephadex LH-20 column chromatography (MeOH) and semipreparative HPLC [Agilent Zorbax SB-C_18_ column, MeOH–H_2_O (containing 0.05% TFA), 40:60, 2 mL/min] to yield **18** (10.5 mg, *t*_R_ = 45.494 min). Fraction A2 was separated on an RP-18 silica gel column eluted with MeOH–H_2_O (20% → 100%) to yield one main fraction. The 40% MeOH-eluted portion was purified by Sephadex LH-20 column chromatography (MeOH) to give three main subfractions, A2-1, A2-2, and A2-3. A2-1 was subjected to silica gel column chromatography eluted with CH_2_Cl_2_ –acetone (20:1) to yield A2-1-1 and A2-1-2. A2-1-1 was isolated by semipreparative HPLC (Agilent Zorbax SB-C_18_ column, MeOH–H_2_O, 42:58, 2 mL/min) to yield **16** (3.6 mg, *t*_R_ = 9.671 min) and **6** (1.6 mg, *t*_R_ = 29.399 min). A2-1-2 was isolated by semipreparative HPLC (Agilent Zorbax SB-C_18_ column, MeOH/H_2_O, 42:58, 2 mL/min) to yield **5** (2.3 mg, *t*_R_ = 14.800 min), **4** (1.4 mg, *t*_R_ = 22.348 min), **3** (1.2 mg, *t*_R_ = 26.288 min), and **1** (0.9 mg, *t*_R_ = 27.934 min). A2-2 was isolated by a silica gel column eluted by CH_2_Cl_2_–MeOH (20:1) and semipreparative HPLC (Agilent Zorbax SB-C_18_ column, MeOH/H_2_O, 45:55, 2 mL/min) to yield **12** (3.0 mg, *t*_R_ = 18.931 min) and **13** (1.7 mg, *t*_R_ = 20.902 min); A2-3 was purified by semipreparative HPLC (Agilent Zorbax SB-C_18_ column, MeOH–H_2_O, 40:60, 2 mL/min) to yield **10** (2.7 mg, *t*_R_ = 19.265 min), **8** (2.7 mg, *t*_R_ = 27.377 min), and **9** (1.4 mg, *t*_R_ = 33.077 min).

Part B was subjected to column chromatography (silica gel; CH_2_Cl_2_/MeOH, 5:1 → 1:1, v/v) to yield two fractions (B1 and B2). Fraction B1 was separated on an RP-18 silica gel column eluted with MeOH/H_2_O (10% → 100%) to yield two main subfractions. The 10% MeOH-eluted portion was purified by Sephadex LH-20 column chromatography (MeOH), silica gel column chromatography (CH_2_Cl_2_/MeOH, 20:1), and semipreparative HPLC (Agilent Zorbax SB-C_18_ column, MeOH/H_2_O, 20:80, 2 mL/min) to yield **2** (11.6 mg, *t*_R_ = 17.869 min). The 20% MeOH eluted part was purified by Sephadex LH-20 column chromatography (MeOH) and semipreparative HPLC Agilent Zorbax SB-C_18_ column, MeOH–H_2_O, 25:75, 2 mL/min) to yield **20** (3.6 mg, *t*_R_ = 26.426 min) and **21** (3.3 mg, *t*_R_ = 28.239 min). Fraction B2 was separated on an RP-18 silica gel column eluted with MeOH-H_2_O (5% → 100%) to yield three main subfractions. The 10% MeOH-eluted part was purified by Sephadex LH-20 column chromatography (MeOH) and silica gel column chromatography (CH_2_Cl_2_–MeOH, 20:1) to yield **23** (2.3 mg) and **19** (4.0 mg). The 30% MeOH-eluted part was purified by Sephadex LH-20 column chromatography (MeOH), silica gel column chromatography (CH_2_Cl_2_–MeOH, 20:1), and semipreparative HPLC (Welch Ultimate AQ-C_18_ column, CH_3_CN–H_2_O, 15:85, 1 mL/min) to yield **7** (2.6 mg, *t*_R_ = 24.990 min). The 40% MeOH-eluted part was purified by Sephadex LH-20 column chromatography (MeOH) and semipreparative HPLC (Agilent Zorbax SB-C_18_ column, MeOH-H_2_O, 40:60, 2 mL/min) to yield **11** (10.5 mg, *t*_R_ = 15.128 min).

### Spectroscopic Data of Compounds

#### (7*S*,8*R*)-9′-*O*-Acetylcedrusin (**1**)

White needles (MeOH); mp 176–179 °C; $\left[ \alpha \right]_{\text{D}}^{23}$ –13 (*c* 0.06, MeOH); UV (MeOH) *λ*_max_ (log*ε*) 306 (3.00), 283 (3.58), 224 (4.02), 204 (4.52) nm; ECD (*c* 0.009, MeOH) *λ*_max_ (Δ*ε*) 294 (+ 0.52), 241 (+ 0.47), 226 (− 0.73), 211 (+ 3.66), 202 (− 2.84) nm; ^1^H and ^13^C NMR data, see Table 1; ESIMS *m*/*z* 411 [M + Na]^+^; HRESIMS *m*/*z* 411.1415 [M + Na]^+^ (calcd for C_21_H_24_NaO_7_, 411.1420).

#### (3*S*,4*S*)-4-Chloro-3-hydroxypiperidin-2-one (**2**)

Light yellow solid; $\left[ \alpha \right]_{\text{D}}^{19}$ –24 (*c* 0.08, MeOH); UV (MeOH) *λ*_max_ (log*ε*) 289 (2.19), 256 (1.92), 197 (3.47) nm; ECD (*c* 0.016, MeOH) *λ*_max_ (Δ*ε*) 221 (− 0.97) nm; ^1^H and ^13^C NMR data, see Table 2; ESIMS *m*/*z* 172 [M + Na]^+^; HRESIMS *m*/*z* 172.0135 [M + Na]^+^ (calcd for C_5_H_8_ClNNaO_2_, 172.0141).

#### 9′-*O*-Acetylisolariciresinol (3)

White solid; $\left[ \alpha \right]_{\text{D}}^{23}$ –6 (*c* 0.09, MeOH); ECD (*c* 0.010, MeOH) *λ*_max_ (Δ*ε*) 217 (− 0.66), 205 (+0.39) nm; ^1^H and ^13^C NMR data, see Table 1; ESIMS *m*/*z* 425 [M + Na]^+^.

### In Vitro Antimicrobial Assays

The bacterial strains, *E. coli* ATCC25922, *S. aureus* subsp. *aureus* ATCC29213, *S. enterica* subsp. *enterica* ATCC14028, and *P. aeruginosa* ATCC27853, and the fungal strain, *C. albicans* ATCC10231, were purchased from China General Microbiological Culture Collection Center. The antimicrobial assays were performed according to modified versions of the CLSI (formerly NCCLS) methods as described previously [43, 44]. Ceftazidime and benzylpenicillin sodium were used as the positive control drugs in the antibacterial assay and amphotericin B was used as the positive control in the antifungal assay.

### ECD Calculations

Computational methods are presented in Supplementary Material.

## Conclusion

Twenty-three compounds including one new lignan, one new lactam, five known lignans, four flavonoid glycosides, and other compounds were isolated from the ethanol extracts of the aerial parts of *L. aequata* collected from Myanmar. 3,4,5-Trihydroxybenzoic acid ethyl ester (**17**) showed the weak inhibitory activities against *E. coli* and *S. enterica* subsp. *enterica*.

## Electronic supplementary material

Below is the link to the electronic supplementary material.

Supplementary material associated with this article (1D and 2D NMR and HRMS spectra of new compounds, chemical structures of known compounds, and computational methods)—Supplementary material 1 (PDF 875 kb)
