# Supplementary material for: Chemical Constituents from Ethanoic Extracts of the Aerial Parts of Leea aequata L., a Traditional Folk Medicine of Myanmar
Source: Nat Prod Bioprospect. 2019 May 7;9(3):243–9. doi: 10.1007/s13659-019-0209-y (PMC6538738; doi:10.1007/s13659-019-0209-y)

Supplementary Material for

Chemical constituents from ethanoic extracts of the aerial part of *Leea aequata* L., a traditional folk medicine of Myanmar

Nay Lin Tun[1,2,3] · Dong-Bao Hu[4] · Meng-Yuan Xia[2] · Dong-Dong Zhang[1,2]· Jun Yang[2] · Thaung Naing Oo[5] · Yue-Hu Wang[2] · Xue-Fei Yang[1,2,3]

✉ Xue-Fei Yang

xuefei@mail.kib.ac.cn

✉ Yue-Hu Wang

wangyuehu@mail.kib.ac.cn

[1] Southeast Asia Biodiversity Research Institute, Chinese Academy of Sciences, Yezin, Nay Pyi Taw 05282, Myanmar

[2] Key Laboratory of Economic Plants and Biotechnology and the Yunnan Key Laboratory for Wild Plant Resources, Kunming Institute of Botany, Chinese Academy of Sciences, Kunming 650201, People's Republic of China

[3] University of Chinese Academy of Sciences, Beijing 100049, People's Republic of China

[4] College of Resources and Environment, Yuxi Normal University, Yuxi, 653100, China

[5] Forest Research Institute, Yezin, Nay Pyi Taw 05282, Myanmar

Contents

Computational methods
**Fig. S1.** Chemical structures of known compounds (**3**–**23**) from *Leea aequata*.
**Fig. S2.** ^{1}H NMR spectrum of **1** (CD_3OD, 800 MHz).
**Fig. S3.** ^{13}C NMR spectrum of **1** (CD_3OD, 201 MHz).
**Fig. S4.** HSQC spectrum of **1**.
**Fig. S5.** ^{1}H–^{1}H COSY spectrum of **1**.
**Fig. S6.** HMBC spectrum of **1**.
**Fig. S7.** ROESY spectrum of **1**.
**Fig. S8.** ECD spectrum of **1.**
**Fig. S9.** HRESIMS spectrum of **1.**
**Fig. S10.** ^{1}H NMR spectrum of **2** (DMSO-d_6, 500 MHz).
**Fig. S11.** ^{13}C NMR spectrum of **2** (DMSO-d_6, 126 MHz).
**Fig. S12.** HSQC spectrum of **2**.
**Fig. S13.** ^{1}H–^{1}H COSY spectrum of **2**.
**Fig. S14.** HMBC spectrum of **2**.
**Fig. S15.** ROESY spectrum of **2**.
**Fig. S16.** ECD spectrum of **2.**
**Fig. S17.** HRESIMS spectrum of **2.**

Computational methods

All DFT and TD-DFT calculations were carried out at 298 K in the gas phase with Gaussian 09.[1] Conformational searches were carried out at the molecular mechanics level of theory employing MMFF force fields.[2-7] The conformers with relative energy within 10 kcal/mol of the lowest-energy conformer were selected and further geometry optimized at the B3LYP/6-311++G(2d,p) level. All the lowest-energy conformers, which correspond to 99% of the total Boltzmann distribution, were selected for ECD spectra calculation. The Boltzmann factor for each conformer was calculated based on Gibbs free energy. Vibrational analysis at the B3LYP/6-311++G(2d,p) level of theory resulted in no imaginary frequencies, confirming the considered conformers as real minima. TDDFT was employed to calculate excitation energy (in nm) and rotatory strength R in dipole velocity form, at the B3LYP/6-311++G(2d,p) level.

References

[1] Gaussian 09.Rev.Cl; Frisch M.J., et al. Gaussian, Inc., Pittsburgh PA, 2009.

[2] Mayer Z.A.; Kallay M.; Kubinyi M.; Keglevich G. *J. M. Strut.* 2009, 906, 94–99.

[3] Silva G.V.J.; Neto A.C. *Tetrahedron* 2005, 61, 7763–7767.

[4] Borkowski E.J.; Suvire F.D.; Enriz R.D. *J. Mol. Struct.* 2010, 953, 83–90.

[5] Zhao S.D.; Shen L.; Luo D.Q.; Zhu H.J. *Curr. Org. Chem.* 2011, 15, 1843–1862.

[6] Li Q.M.; Ren J.; Zhou B.D.; Bai B.; Liu X.C.; Wen M.L.; Zhu H.J. *Tetrahedron* 2013, 69, 3067–3074.

[7] Hu D.B., Li W.X., Zhao Z.Z., Feng T., Yin R.H., Li Z.H., Liu J.K., Zhu H.J. *Tetrahedron Lett.* 2014, 55, 6530–6533.

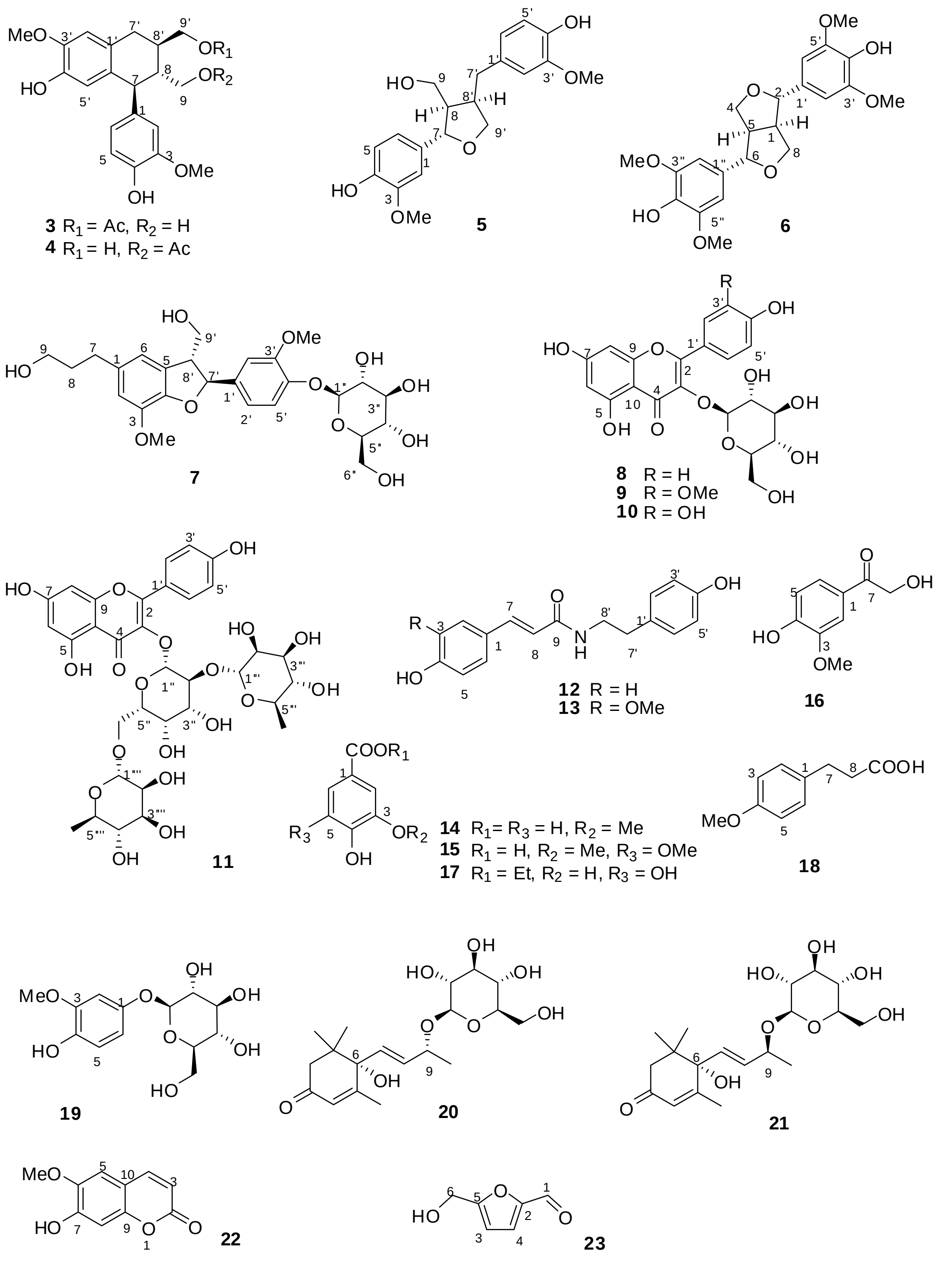

Fig. S1. Chemical structures of known compounds (**3**–**23**) from *Leea aequata*.

Fig. S2. ^{1}H NMR spectrum of **1** (CD_3OD, 800 MHz).

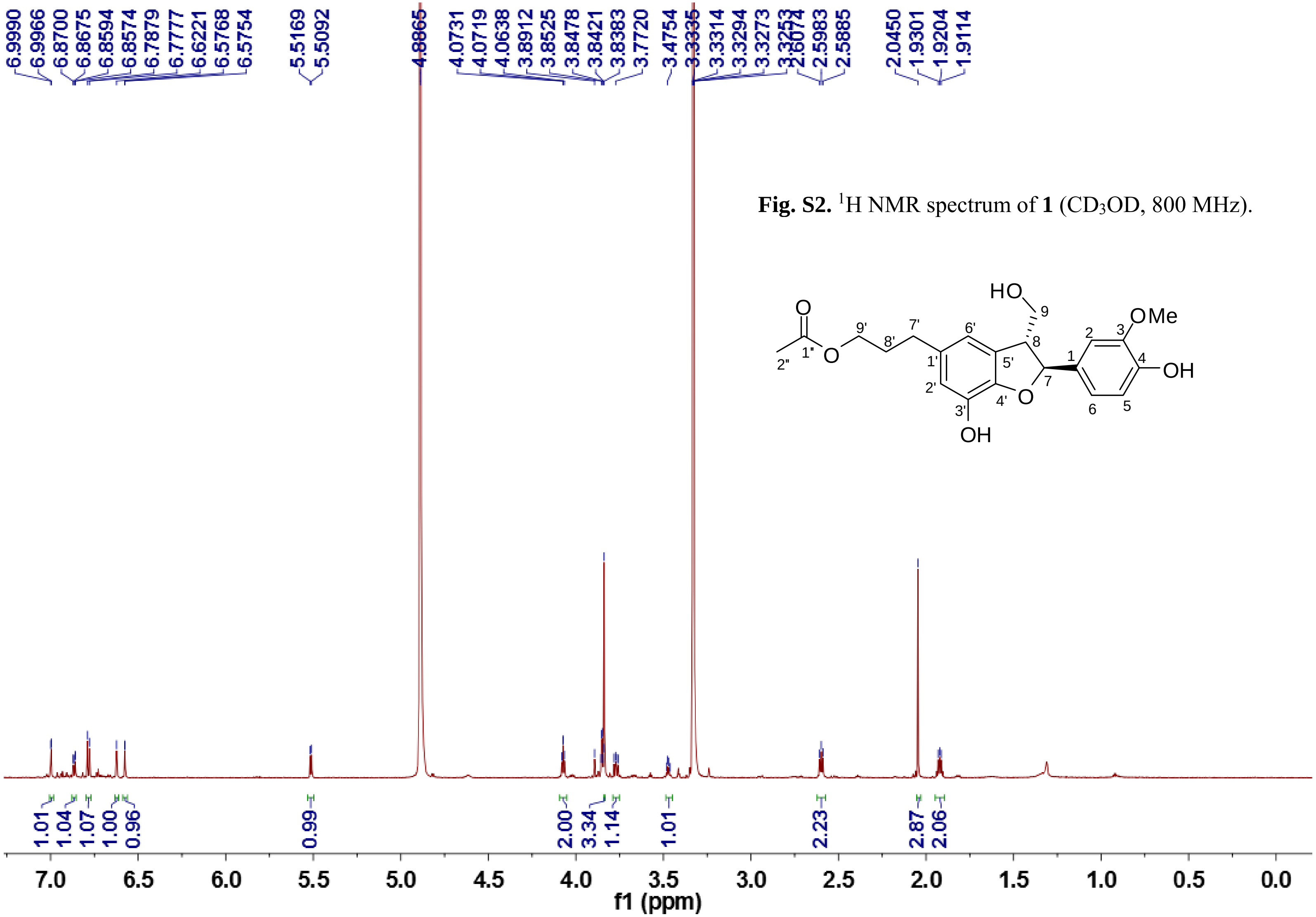

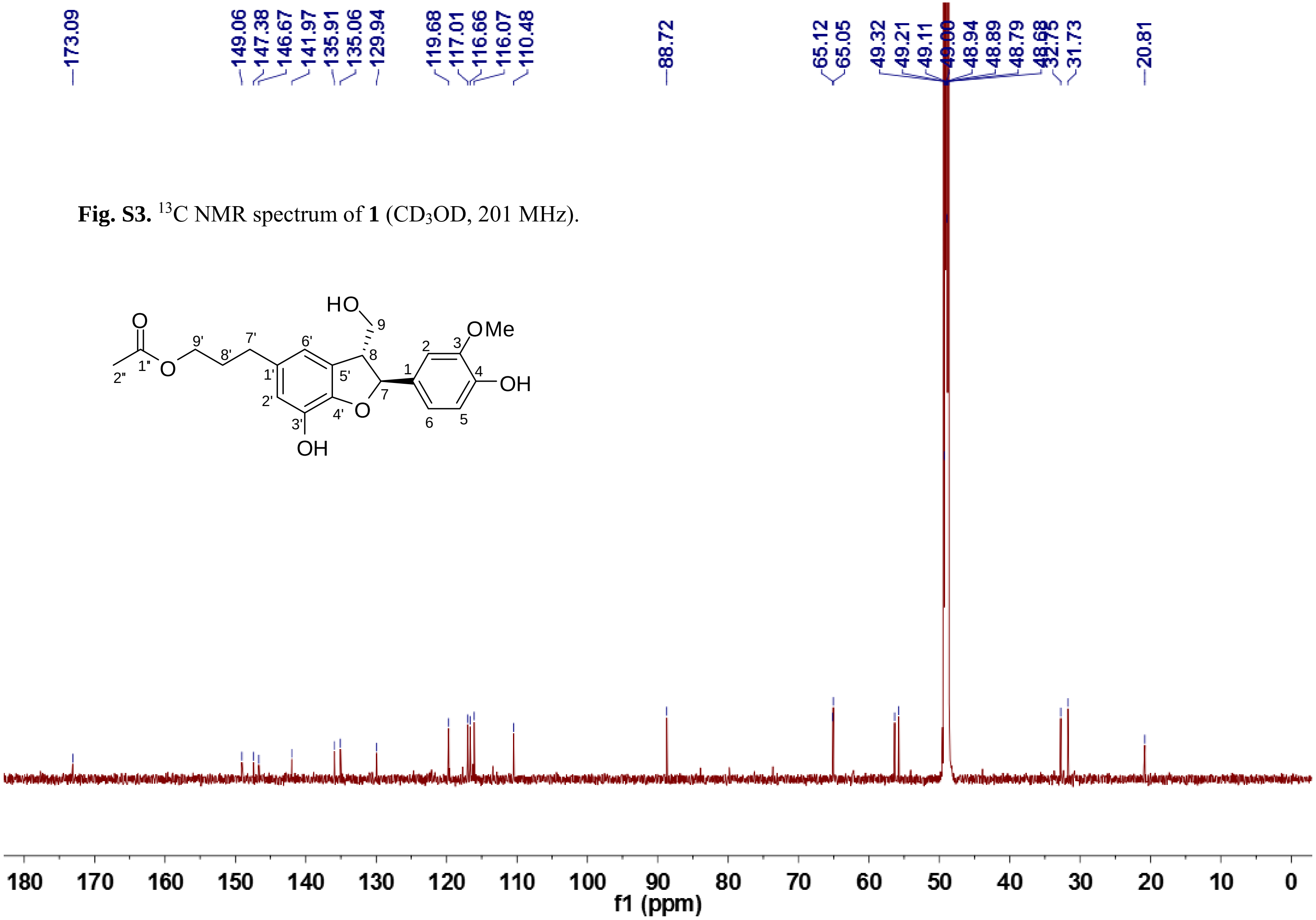

Fig. S3. ^{13}C NMR spectrum of **1** (CD_3OD, 201 MHz).

Fig. S4. HSQC spectrum of **1**.

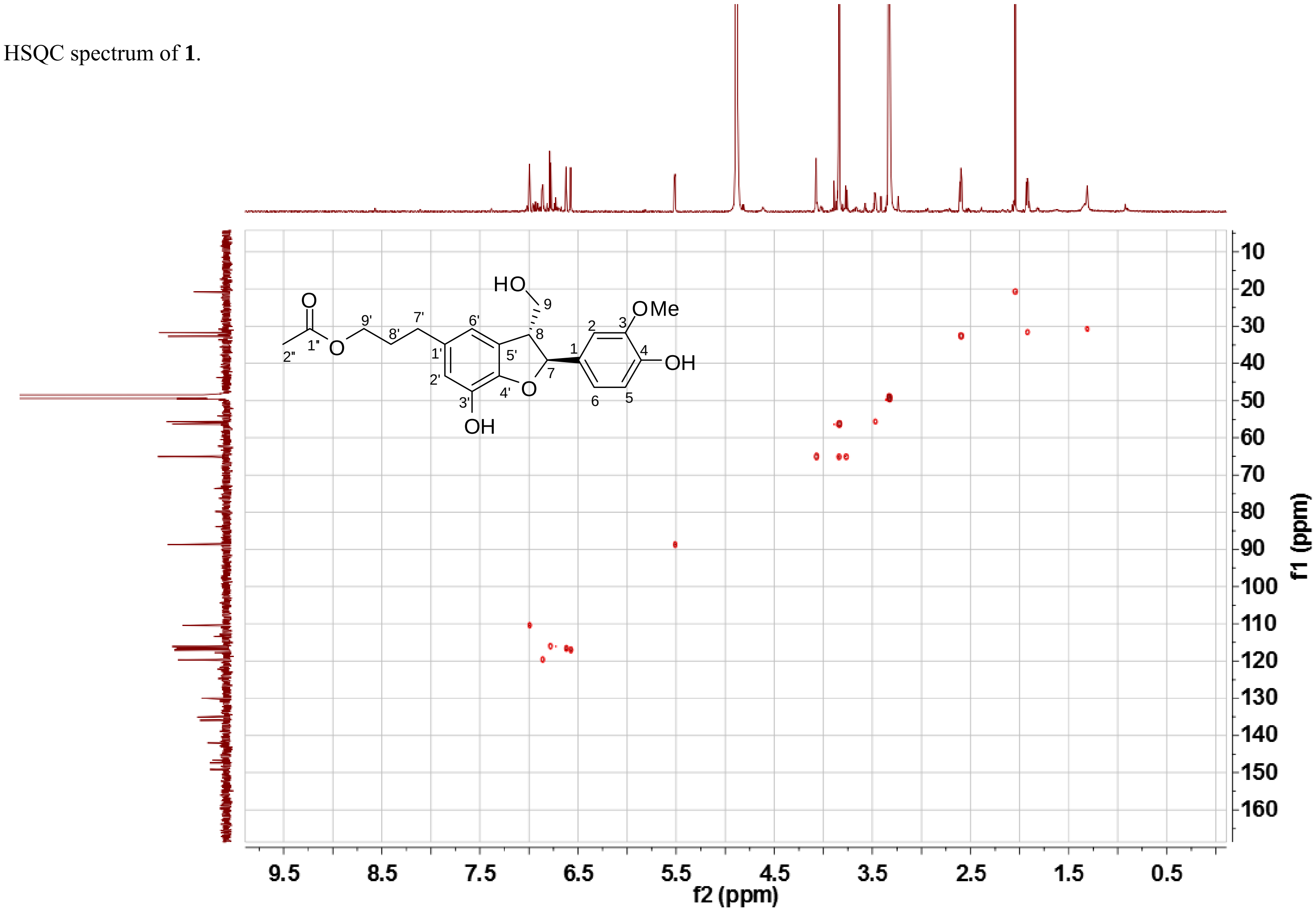

Fig. S5. $^1H–^1H$ COSY spectrum of **1**.

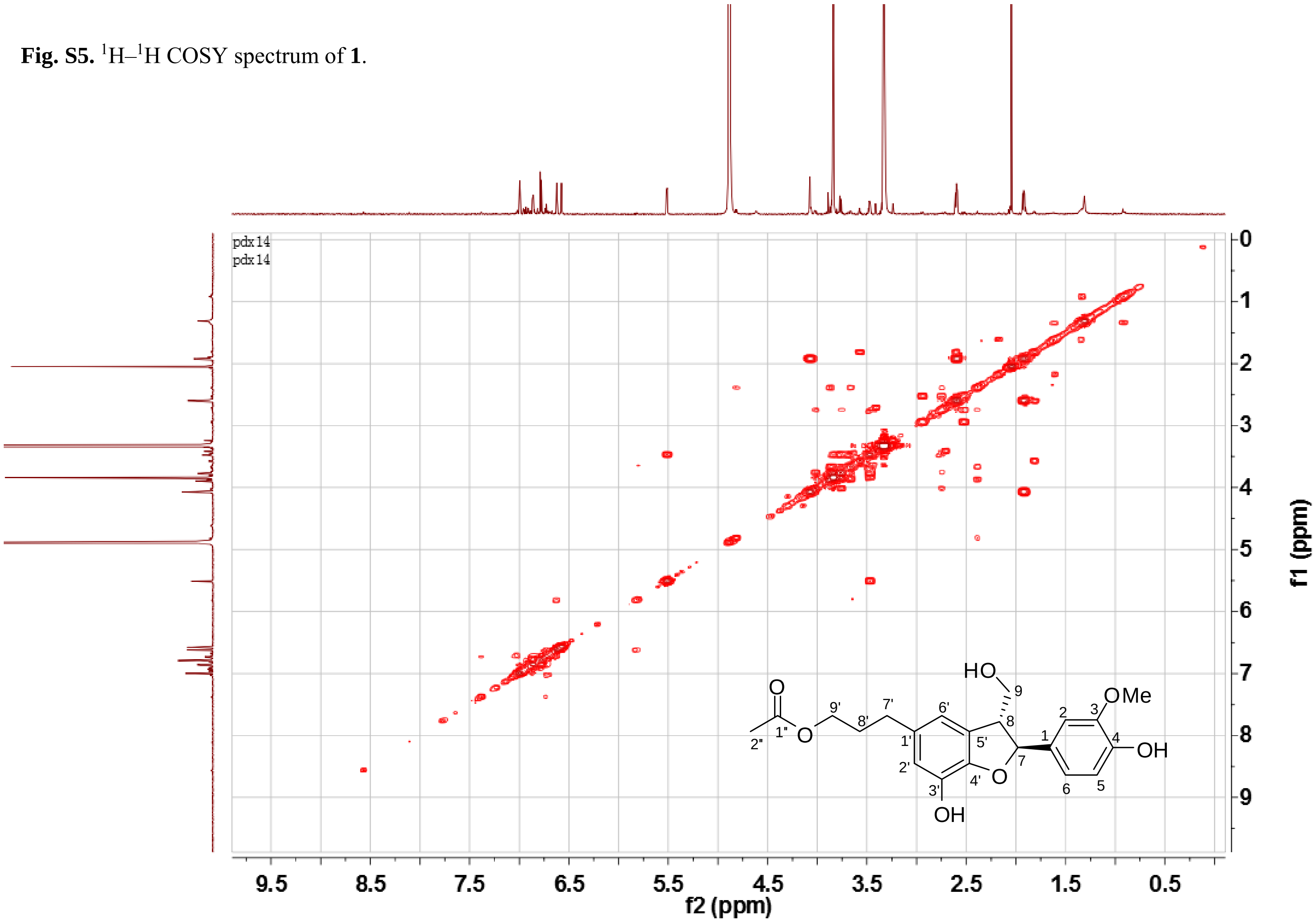

Fig. S6. HMBC spectrum of **1**.

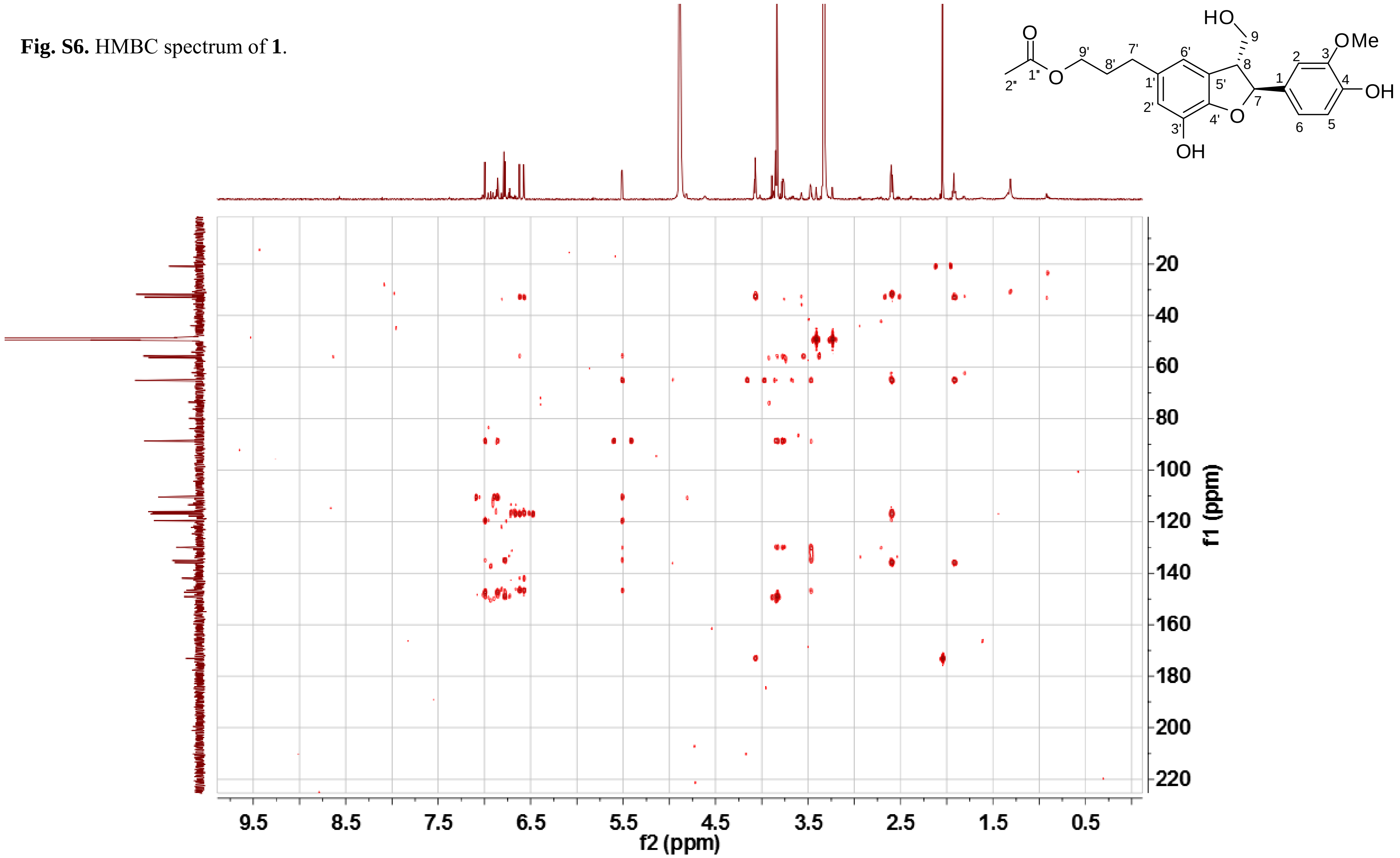

Fig. S7. ROESY spectrum of **1**.

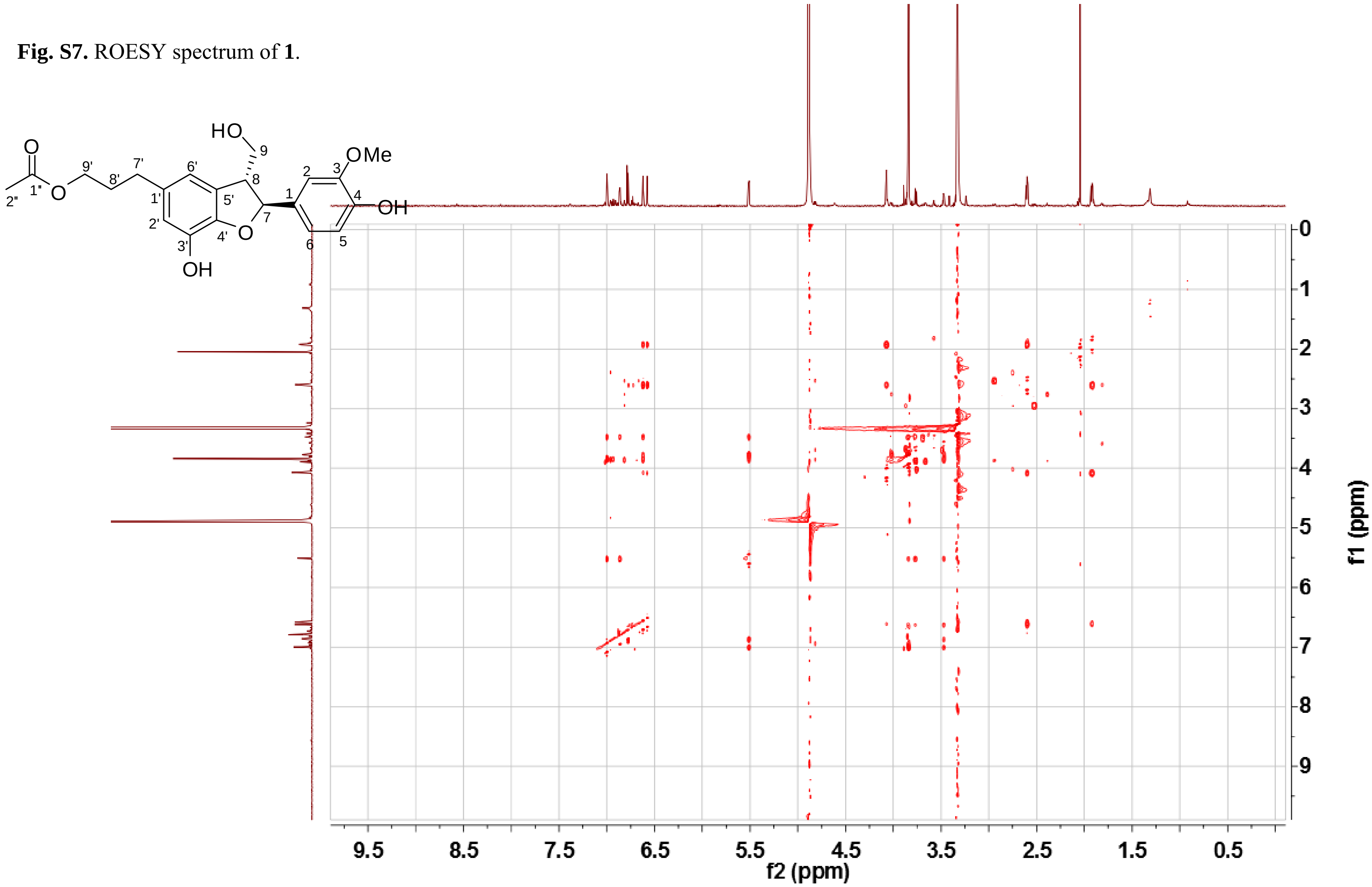

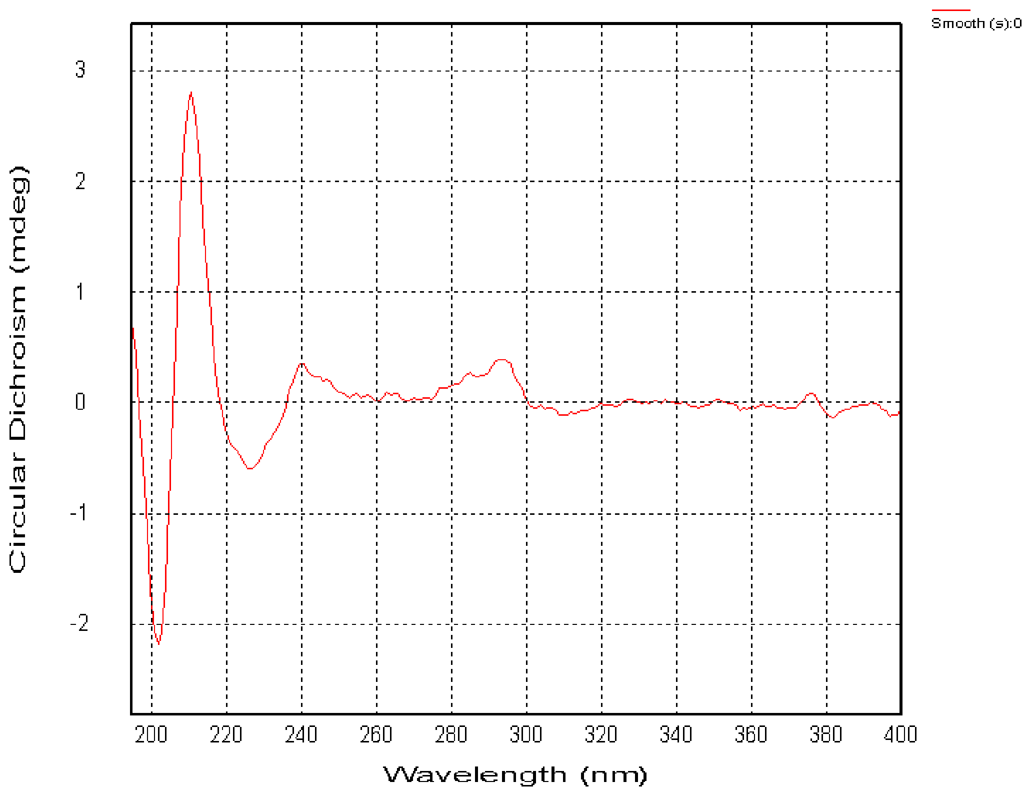

File: CD PDX14-1mm(195-400)18062910.dsx
ProBinaryX
Attributes :
- Time Stamp :Fri Jun 29 15:43:54 2018

- File ID : {6443DCA7-BB07-45c1-BF99-7F9313AC8AEA}
- Is CFR Compliant : false
- Original data has not been modified.

Remarks:
- User: APLService
- Date: 2018/06/29
- Instrument: 0218
- DetectorType: PMT
- DichOS Calibration Correction Curve: 0218/1
- HV (CDDC channel): 0 v
- Time per point: 1 s
- Description: Sample 1
- Concentration: 0.0900mg/mL MeOH
- Pathlength: 1 mm
- Temperature: 20℃

Settings:
- Time-per-point: 1s (25us x 40000)
- SE
- Wavelength: 195nm - 400nm
- Step Size: 1nm
- Bandwidth: 1nm

Fig. S8. ECD spectrum of **1.**

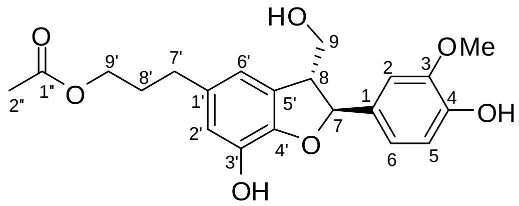

Comment

**Sample Group**		**Info.**
**Acquisition SW**	6200 series TOF/6500 series	
**Version**	Q-TOF B.05.01 (B5125.2)	

User Spectra

Fragmentor Voltage	Collision Energy	Ionization Mode
240	0	ESI

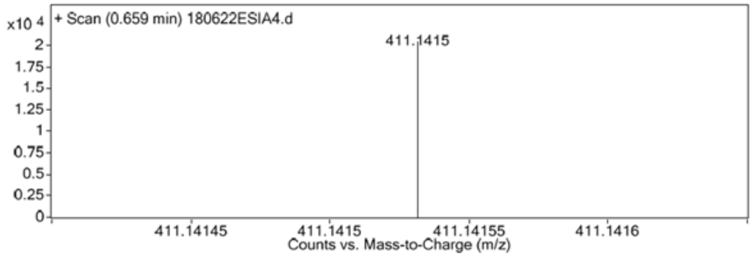

Peak List

m/z	z	Abund
256.2634	1	56791.98
274.274	1	236106.63
302.3053	1	66859
318.3001	1	71351.05
437.1947	1	365283.41
438.1976	1	91160.72
453.1681	1	49052.91
512.5041	1	60031.68
591.4962	1	47341.98
619.5277	1	79607.68

Formula Calculator Element Limits

Element	Min	Max
C	0	200
H	0	400
O	0	10
Na	1	1

Formula Calculator Results

Formula	CalculatedMass	Mz	Diff.(mDa)	Diff. (ppm)	DBE
C21 H24 Na O7	411.1420	411.1415	0.5	1.1	9.5

--- End Of Report ---

Fig. S9. HRESIMS spectrum of **1.**

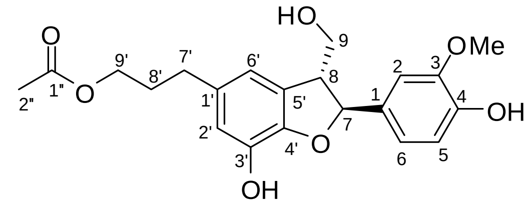

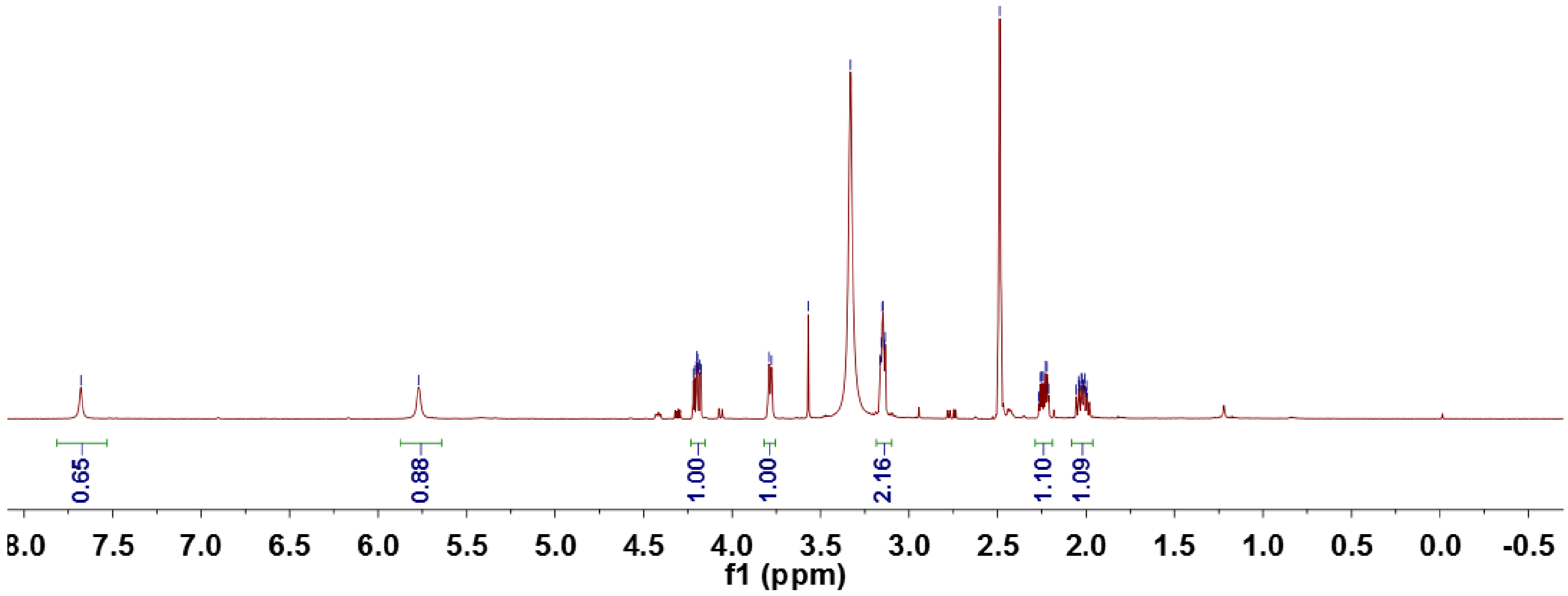

Fig. S10. ¹H NMR spectrum of **2** (DMSO-d_6, 500 MHz).

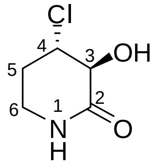

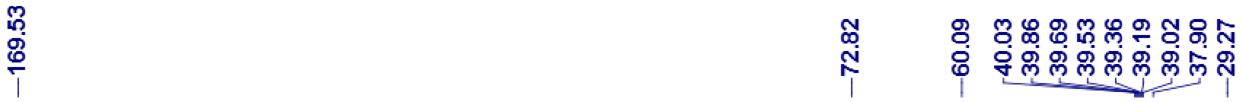

Fig. S11. ^{13}C NMR spectrum of **2** (DMSO-d_6, 126 MHz).

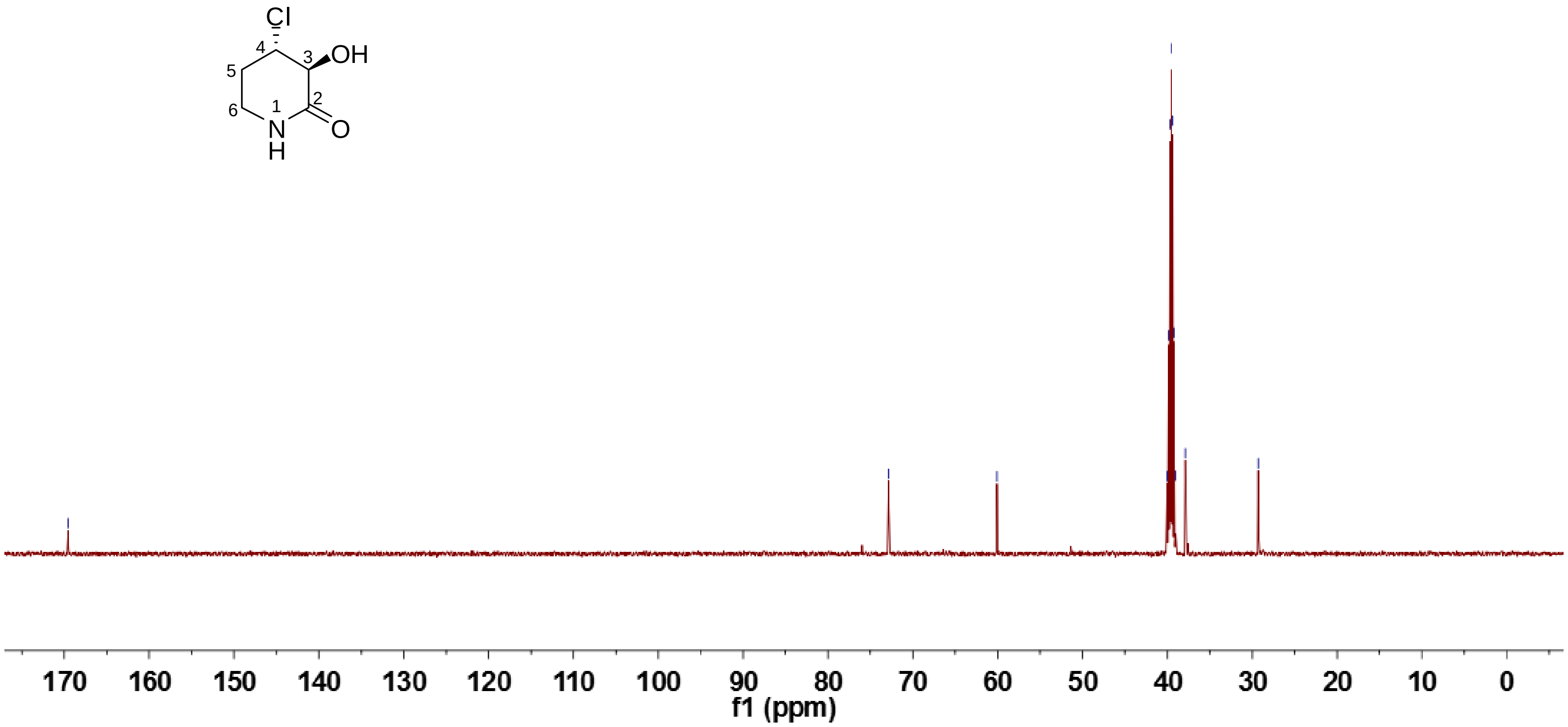

Fig. S12. HSQC spectrum of **2**.

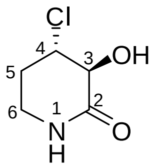

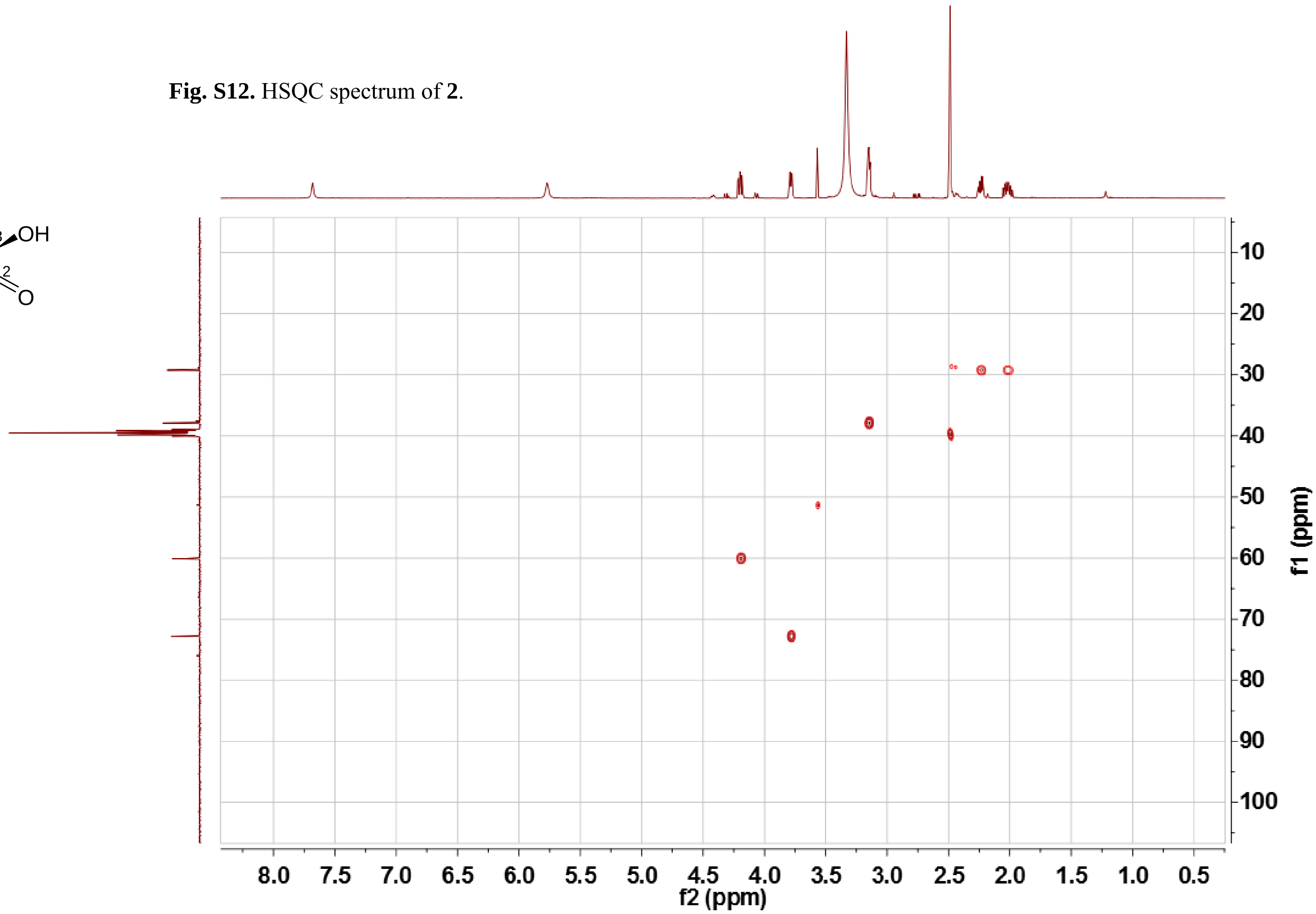

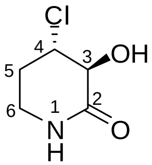

Fig. S13. ^{1}H–^{1}H COSY spectrum of **2**.

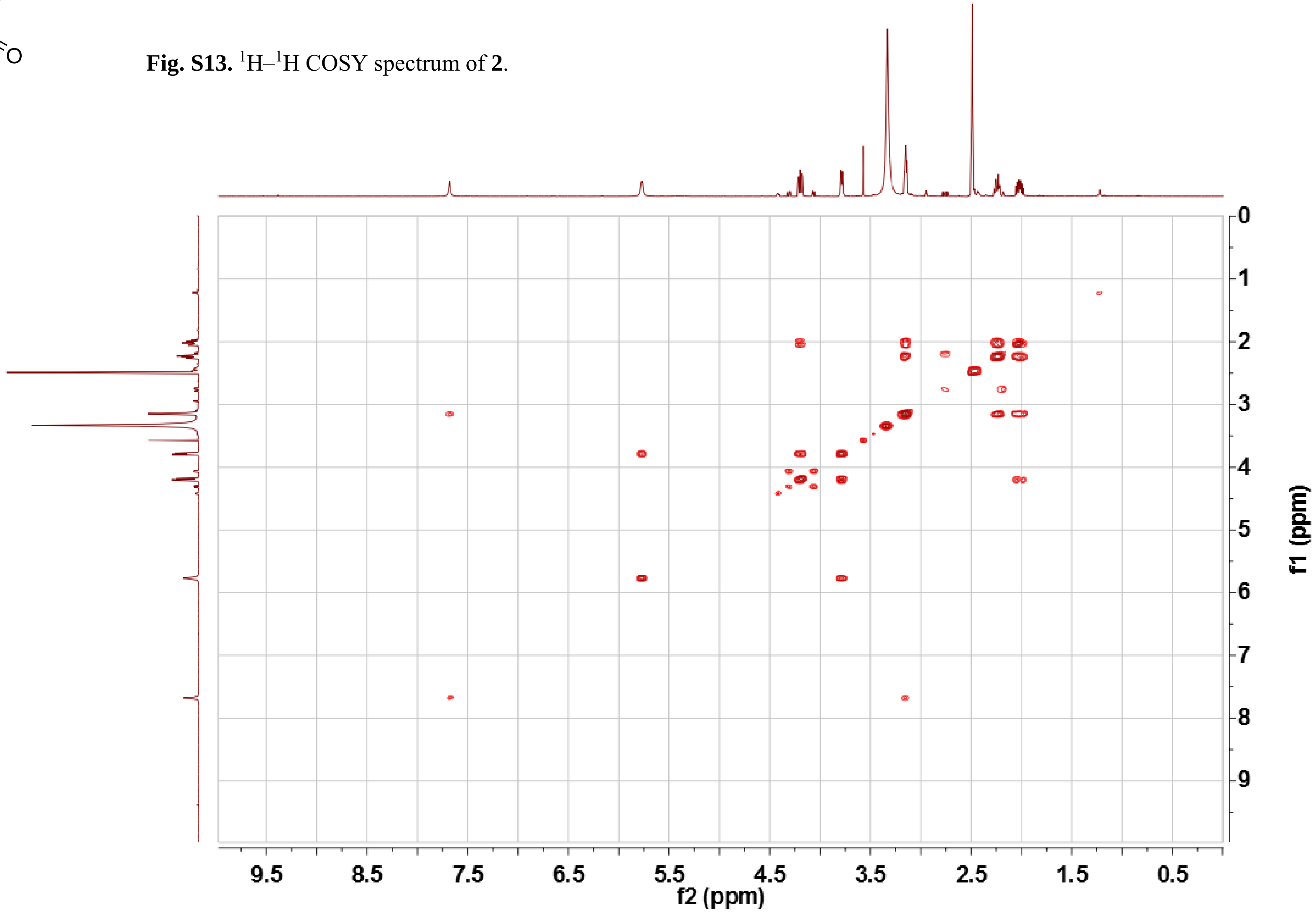

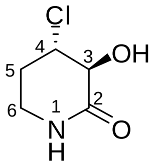

Fig. S14. HMBC spectrum of **2**.

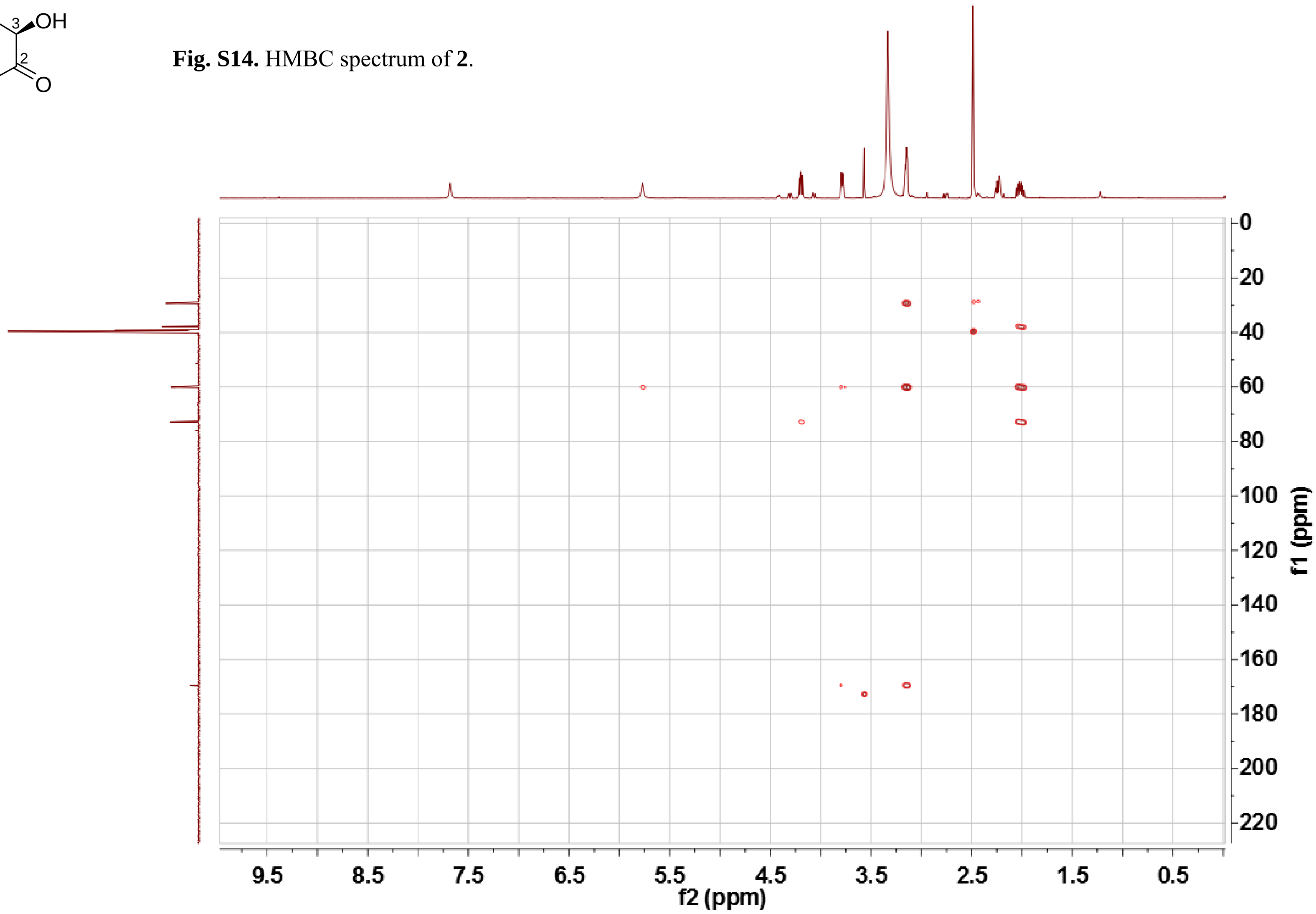

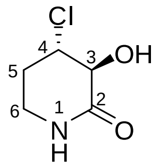

Fig. S15. ROESY spectrum of **2**.

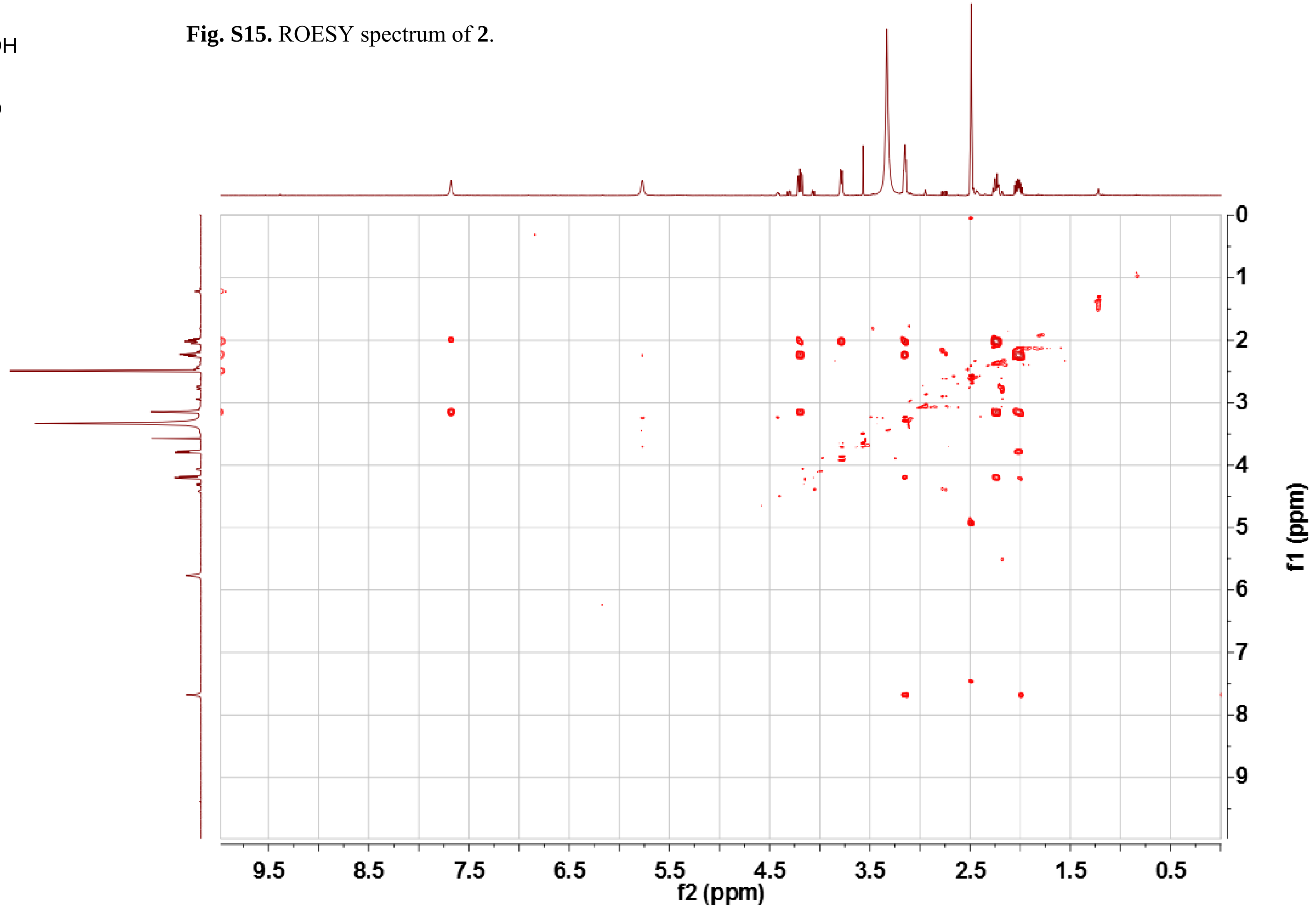

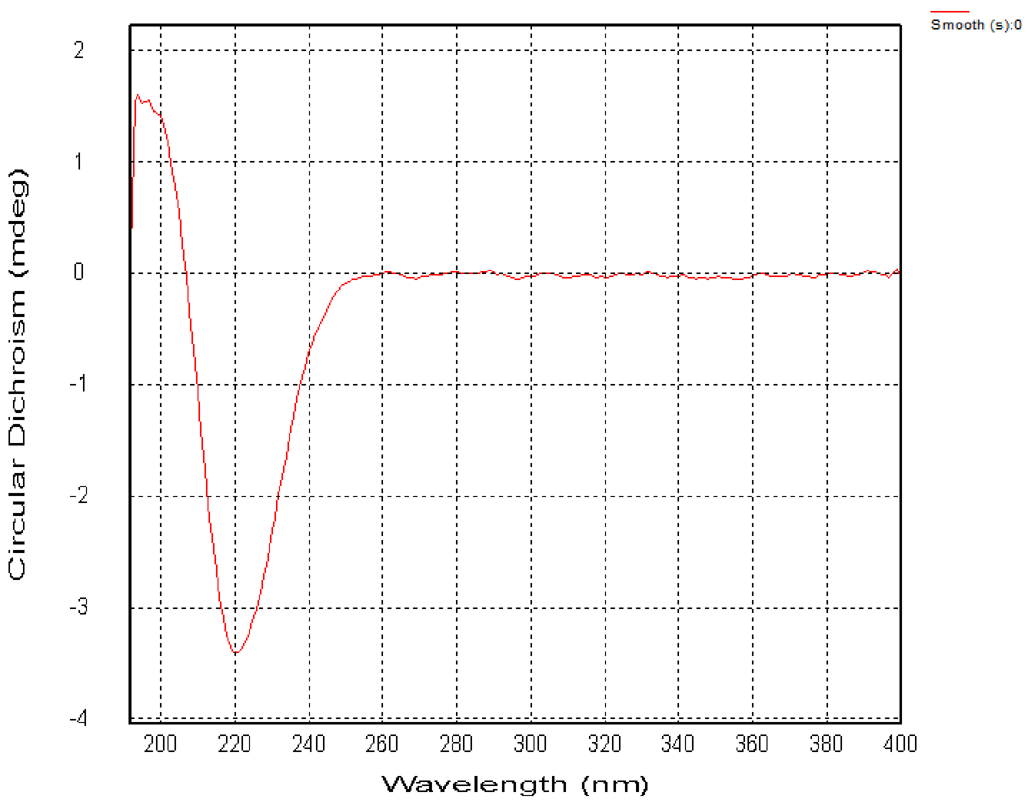

Fig. S16. ECD spectrum of **2.**

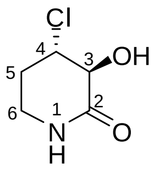

File: pdx3800670.dsx
ProBinaryX
Attributes :
- Time Stamp :Tue Jan 08 16:03:47 2019

- File ID : {0BAE3DBE-C93C-4603-85F1-FC49F739AE57}
- Is CFR Compliant : false
- Original data has not been modified.

Remarks:
- User: CD
- Date: 2019/01/08
- Instrument: 0547
- DetectorType: LAAPD
- DichOS Calibration Correction Curve: 0547/2
- HV (CDDC channel): 0 v
- Time per point: 0.25 s
- Description: pdx38
- Concentration: 0.16 mg/ml CH_3OH
- Pathlength: 1 mm
- Temperature: ---- C

Settings:
- Time-per-point: 0.25s (25us x 10000)
- SE
- Wavelength: 192nm - 400nm
- Step Size: 1nm
- Bandwidth: 1nm
- 3 repeats in set.
- -iter option selected

**Data Filename**	190109ESIA1.d	**Sample Name**	pdx38
**Sample Type**	Sample	**Position**	
**Instrument Name**	Agilent G6230 TOF MS	**User Name**	KIB
**Acq Method**	ESI.m	**Acquired Time**	1/8/2019 3:38:07 PM
**IRM Calibration Status**	Success	**DA Method**	ESI.m
**Comment**			

**Sample Group**		**Info.**
**Acquisition SW Version**	6200 series TOF/6500 series Q-TOF B.05.01 (B5125.2)	

User Spectra

Fragmentor Voltage	Collision Energy	Ionization Mode
150	0	ESI

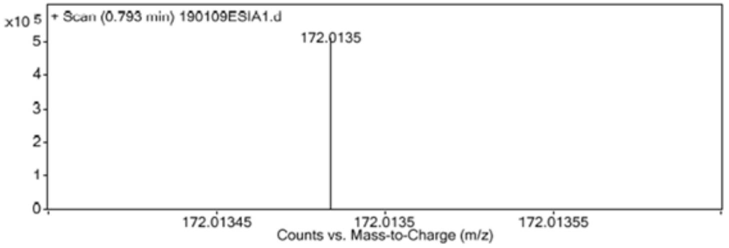

Peak List

m/z	z	Abund	Formula	Ion
121.0509		96027.35		
172.0135	1	507811.41	C5 H8 Cl N Na O2	M+
174.0106	1	161641.61	C5 H8 Cl N Na O2	M+
196.0574		96870.79		
213.0402	1	312443.41		
215.0401	1	101590.24		
321.0382	1	141984.47		
323.0359	1	87882.53		
618.0706	1	79125.71		
620.0682	1	86036.02		

Formula Calculator Element Limits

Element	Min	Max
C	0	200
H	0	400
O	0	10
Na	1	1
N	1	1
Cl	1	1

Formula Calculator Results

Formula	CalculatedMass	Mz	Diff.(mDa)	Diff. (ppm)	DBE
C5 H8 Cl N Na O2	172.0141	172.0135	0.6	3.7	1.5

--- End Of Report ---

Fig. S17. HRESIMS spectrum of **2.**

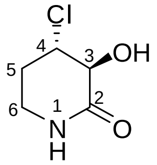

Supplement: Supplementary file 1 — Supplementary material associated with this article (1D and 2D NMR and HRMS spectra of new compounds, chemical structures of known compounds, and computational methods)—Supplementary material 1 (PDF 875 kb) [file 13659_2019_209_MOESM1_ESM.pdf]
